# Synthesis of magnetic Fe_3_O_4_/graphene aerogel for the removal of 2,4-dichlorophenoxyacetic acid herbicide from water

**DOI:** 10.1039/d4ra03567d

**Published:** 2024-07-15

**Authors:** Thu Hang Thi Nguyen, Kim Thuy Nguyen, Bao Hung Le, Xuan Truong Nghiem, Duc Duong La, Duy Khiem Nguyen, Hoai Phuong Thi Nguyen

**Affiliations:** a Department of Chemistry and Environment, Joint Vietnam-Russia Tropical Science and Technology Research Center 63 Nguyen Văn Huyen Ha Noi Vietnam hoaiphuong1978@gmail.com; b Institute of Chemistry & Materials Science 17 Hoang Sam Hanoi Vietnam; c Center for Advanced Chemistry, Institute of Research and Development, Duy Tan University 03 Quang Trung Da Nang Vietnam; d Faculty of Natural Sciences, Duy Tan University 03 Quang Trung Da Nang Vietnam

## Abstract

Graphene-based aerogels are among the lightest materials in the world and have been extensively studied for environmental remediation. In this work, an Fe_3_O_4_/graphene aerogel material was synthesized using the co-precipitation method. The prepared material was characterized using X-ray diffraction (XRD), scanning electron microscopy/X-ray energy dispersive spectroscopy (FESEM/EDX), infrared spectroscopy (FT-IR), and vibration sample magnetization (VSM). The results showed that the Fe_3_O_4_ nanoparticles with a particle size of less than 100 nm were well-distributed on the surface of the graphene aerogel. The prepared Fe_3_O_4_/graphene aerogel showed effective removal of 2,4-D herbicide from the aqueous solution with a maximal adsorption capacity of approximately 42.918 mg g^−1^. The adsorption isotherms and kinetics were investigated to study the adsorption behaviour of the resultant material. The saturation magnetism value of the aerogel was determined to be about 20.66 emu g^−1^, indicating that the adsorbent could be easily collected from the solution using an external magnet. These results implied that the prepared Fe_3_O_4_/graphene aerogel could be a promising adsorbent for the removal of 2,4-D herbicide from water.

## Abstract 2

Graphene-based aerogels are among the lightest materials in the world and have been extensively studied for environmental remediation.

## Graphical abstract

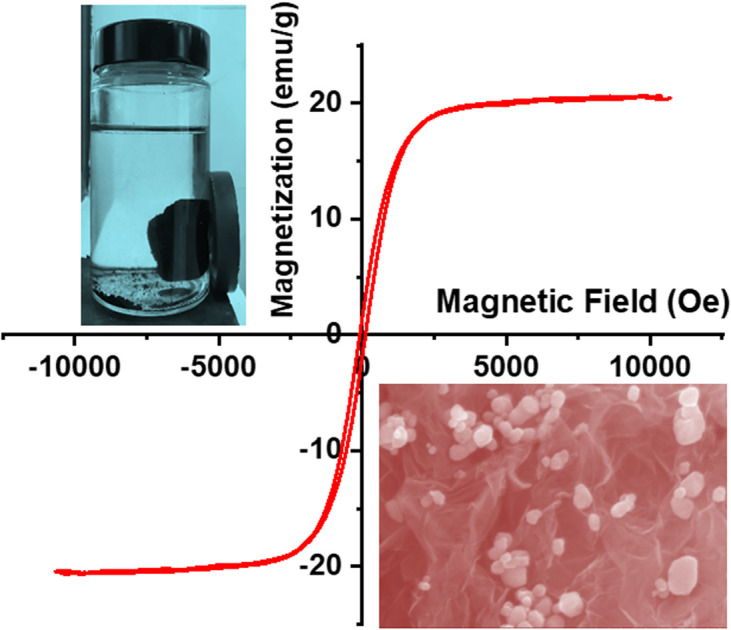

## Introduction

There is no naturally occurring 2,4-dichlorophenoxyacetic acid (2,4-D) in the environment. Herbicide 2,4-D is extensively utilized both domestically and internationally, serving as the primary component in numerous formulations.^1–3^ Its purpose is to effectively eliminate weeds on land and in aquatic environments.^4,5^ The substance is a colorless to light brown solid with no smell, and it has a higher density than water, causing it to sink. 2,4-D is a chlorophenoxyacetic acid with chlorine substituting the ring hydrogens at positions 2 and 4. It functions as a synthetic auxin, a substance that mimics the effects of a plant hormone, as well as a defoliant, an agrochemical (a chemical used in agriculture), an EC inhibitor (explicitly inhibiting the enzyme shikimate dehydrogenase), an environmental pollutant, and a phenoxy herbicide (a type of herbicide that contains the phenoxy group).^4,6–9^ 2,4-D is a widely used herbicide that acts throughout the entire plant system and is effectively manages broadleaf weeds.^8,10^ It is the most extensively employed herbicide globally and the third most frequently utilized in North America.^11,12^ Often used as an addition to plant cell culture media like MS medium and in laboratory settings for plant research, 2,4-D is a notable artificial auxin.^13,14^ This herbicide induces ocular irritation and gastrointestinal distress, and has the potential to be hazardous to fish and other aquatic species.^15–17^ 2,4-D was a constituent of Agent Orange, the pesticide that was extensively utilized during the Vietnam War. While 2,4-D accounted for 50% of the composition of Agent Orange, the health consequences of Agent Orange are mainly attributed to the presence of dioxin pollutants produced during its manufacturing process rather than 2,4-D itself. Methods have been studied to remove 2,4-dichlorophenoxyacetic acid, including degradation,^18,19^ adsorption,^20–24^ filtration,^25^ electrochemically assisted adsorption,^26^ biodegradation,^27–29^*etc.*

Due to its exceptional electrical, mechanical, and other properties, graphene – a two-dimensional thick carbon atom organized in a honeycomb lattice – has drawn a lot of interest for its possible uses in sensors,^30–33^ catalysis,^34–37^ energy storage devices,^38–42^ and the environment. In general, strong oxidants can oxidize graphene, which can then be readily peeled off by reducing agents to generate graphene oxide (GO) and reduced graphene oxide (rGO).^43–46^ Multiple oxygen-containing functional groups are produced in GO and rGO by chemical oxidation modification techniques, offering a possible low-cost method for large-scale manufacturing of graphene-based products. Graphene's distinct surface characteristics make it a perfect platform for hybridizing nanoparticles in a variety of applications, such as lithium-ion batteries,^47–50^ environment,^51–53^ and agriculture.^54–56^ In the environment, metal oxide nanoparticles were combined with graphene as a framework to enhance their adsorption capabilities for sizes up to micrometers and thicknesses below a few nanometers.^57–60^ Aerogels based on graphene are among the world's lightest materials. Because of their remarkable qualities, which include high mechanical strength, electrical conductivity, thermal resistance, and adsorption capacity, the academy and industry are becoming increasingly interested in them, consequently, research was done on the intriguing prospective uses of graphene aerogels in energy storage, energy conversion, and environmental preservation.^61–64^ Through adsorption or photocatalytic degradation, 2,4-dichlorophenoxyacetic acid is eliminated using graphene-based composites.^65–68^ However, the aerogel used to treat water environments is difficult to recover. Scientists have researched ways to change the properties or state of existence of composites derived from graphene aerogel, such as by forming films, fibers, sheets, *etc.*,^69–71^ or magnetizing the material.^72–74^ Magnetic Fe_3_O_4_ is combined with the initial materials to create magnetization for the material. Magnetic aerogel composites have a saturation magnetization of 20–100 emu g^−1^.^72,74–76^ The magnetic material is quickly recovered and reused after removing toxic compounds from the water.

In this work, co-precipitation and partial reduction approaches were utilized to fabricate magnetic Fe_3_O_4_/graphene aerogel composite. Nanocomposites have been used to remove pesticide 2,4-dichlorophenoxyacetic acid from aqueous solution.

## Materials and methods

### Materials

Without any extra purification, all compounds were used exactly as they were supplied. Graphite flakes ≥99.5%, C_6_H_8_O_6_ 99.0–110.5%, NaOH ≥97.0%, acetic acid 100% were obtained from Merck (Germany). Chemicals such as acetone nitrile ≥99.9%, 2,4-dichlorophenoxyacetic acid 97%, FeCl_3_·6H_2_O 99.0%, and FeCl_2_·4H_2_O 98.0% were purchased from Sigma Aldrich (USA).

### Preparation of graphene oxide

Using the enhanced Hummer process, graphite was converted into graphene oxide (GO) as follows: After adding 3 g of graphite powder to 42 mL of concentrated H_2_SO_4_, cool the mixture to 0 and 5 °C and stir consistently for 30 minutes. 0.45 g of KMnO_4_ was gradually added to the reaction mixture and agitated continuously for 15 minutes. After that, the mixture was agitated for 30 minutes while the temperature was kept at no higher than 35 °C and 9 g of KMnO_4_ was added. The combination is gradually stirred, distilled water is added, and the system temperature is kept at or below 50 °C for an hour. 10.5 mL of 30% H_2_O_2_ were added following the reaction in order to eliminate any leftover manganese dioxide and permanganate. After filtering, the substance was cleaned to a pH of 7 using distilled water and diluted HCl acid. The product was vacuum-dried at 60 °C. Using an agate mortar, the dried substance was ground fine and put in a sealed jar for storage.^77^

### Preparation of magnetic Fe_3_O_4_/graphene aerogel (Fe_3_O_4_/GA)

Disperse 0.8 g GO into 95 mL of a solution mixture containing 1 mM FeCl_3_·6H_2_O and FeCl_2_·4H_2_O. Stir the mixture in the induction cooker while slowly adding 2 M NaOH solution until pH ∼ 10. Ultrasound the system for 30 minutes and heat to about 80 °C. Add 0.8 g C_6_H_8_O_6_, and continue sonication for 20 minutes. To create Fe_3_O_4_/graphene hydrogel (Fe_3_O_4_/GH), transfer the mixture to a thermos and heat it at 90 °C for six hours. To obtain Fe_3_O_4_/GA material, sample Fe_3_O_4_/GH was refrigerated at −30 °C for 6 hours and then freeze-dried at −50 °C for 48 hours.

### Characterization

Applying an X'Pert Pro X-ray diffractometer (XRD) with a CuKα anode and a 2*θ* range of 10° to 70°, the phase of the material was closely analyzed at a rate of 5° min^−1^. The morphology of Fe_3_O_4_/GA was investigated employing scanning electron microscopy (SEM). Fe_3_O_4_/GA was calculated using energy-dispersive X-ray spectroscopy (EDX). The porosity of the materials was comprehensively examined at 77 K using the nitrogen gas adsorption isotherm (BET). FTIR infrared spectroscopy was employed to investigate the material's bonds and functional groups in the 400–4000 cm^−1^ wavenumber region. Magnetic force on a VSM device with a range of −10 000 to 10 000 Oe was used to assess magnetic saturation.

### Adsorption of 2,4-dichlorophenoxyacetic acid

A constant amount of Fe_3_O_4_/GA (0.02 g) was added to 20.0 mL of an aqueous solution containing a predefined amount of 2,4-D in order to conduct batch adsorption testing. All of the test parameters, adsorbent concentration, nanocomposite dosage, contact time, and pH, were varied while the experiments were carried out at room temperature (27 °C). The adsorption isotherm and kinetics were measured at 160 rpm and 30 C at predetermined intervals while the flasks were being agitated in a circular shaker. After the adsorption procedure is complete, filter the mixture and use high-performance liquid chromatography on the Agilent Technologies of America 6340 Triple Quad LC/MS to measure the 2,4-D content. Sample measurement conditions were established: Zorbax Eclipse Plus C18 column 2.1 × 50 mm; *V*_sample_: 1.8 μm; mobile phase solvent A: 0.05% acetic acid, B: acetonitrile; flow rate: 0.26 mL min^−1^.

The following formulas were used to determine the material's 2,4-D adsorption capacity and removal efficiency:
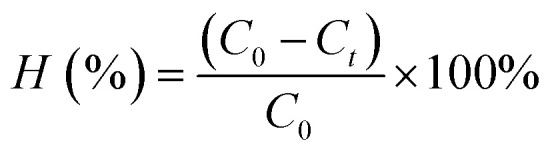

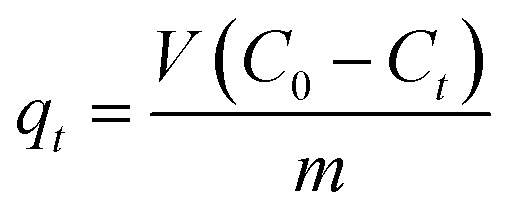
where: *V* is the volume of 2,4-D solution (L); *C*_0_, *C*_*t*_ are the concentrations of before and after treatment 2,4-D solutions, respectively (mg L^−1^); *m* is the mass of Fe_3_O_4_/GA (g).

#### Effect of time

Adsorb 20 mL of 2,4-D solution at a concentration of 100 mg L^−1^ with 0.02 g of Fe_3_O_4_/GA material shaking at 30, 60, 120, 150, 180, 210, 240, and 300 minute intervals.

#### Effect of pH

In general, a solution's pH has a significant impact on the physicochemical processes occurring at the water–solid interface. The effect of pH on 2,4-D adsorption capacity was examined using 0.02 g of Fe_3_O_4_/GA material adsorbing 20 mL of 50 mg L^−1^ 2,4-D solution at various pH values (3, 4, 5, 6, 7, 8, 10). The pH was adjusted using solutions of 1 M HCl and 1 M NaOH.

#### Effect of initial concentration

The adsorption effectiveness of the adsorbents is influenced by the initial concentration of the simulated dye solution. 20 mL of 2,4-D solution and 0.02 g of Fe_3_O_4_/GA were mixed at starting concentrations of 10, 30, 50, 70, 90, and 110 mg L^−1^ in order to study adsorption.

### 2,4-D adsorption isotherm of Fe_3_O_4_/GA

The adsorption of 2,4-D on Fe_3_O_4_/GA has been studied using a variety of adsorption isotherm models include Langmuir, Freundlich, Temkin, and Dubinin–Radushkevich (Table 1).

**Table 1:** The isotherm models' linear equations

Isotherm model	Equation
Langmuir	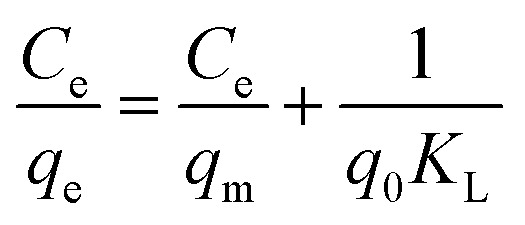
Freundlich	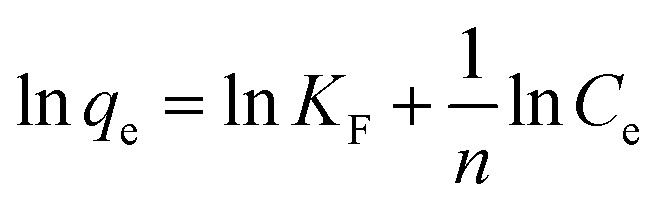
Temkin	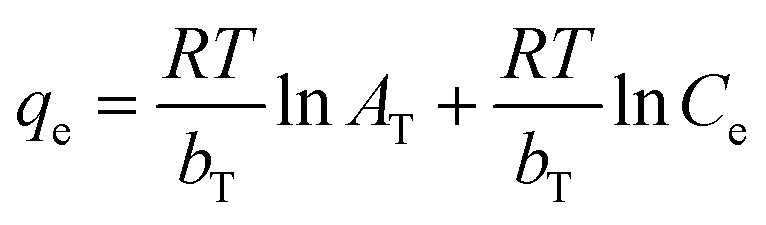
Dubinin–Radushkevich	ln *q*_e_ = ln *q*_m_ − *βε*^2^

## Results and discussion

**Fig. 1:**
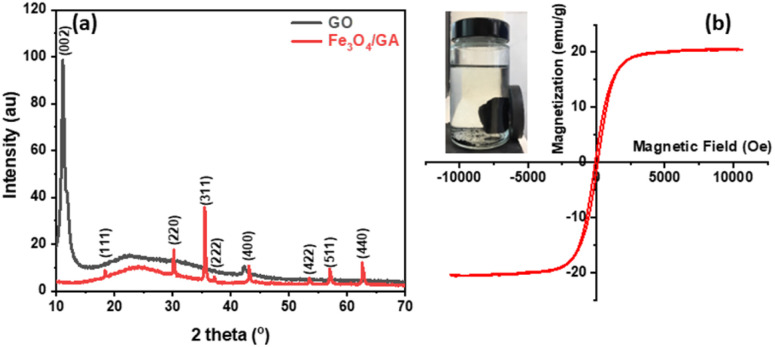
The XRD diffraction pattern (a) and hysteresis curve (b) of GO and Fe_3_O_4_/GA materials.

The findings of the X-ray diffraction analysis of the generated materials are displayed in Fig. 1a. The sharp diffraction peak (002) at 2*θ* = 11.16° is linked to GO.^78,79^ The XRD patterns of GO and Fe_3_O_4_/GA are shown in Fig. 1. The peaks at 2𝜃 have values of 18.333° (111), 30.158° (220), 35.522° (311), 37.158° (222), 43.173° (400), 53.563° (422), 57.100° (511), 62.704° (440) confirmed the formation of Fe_3_O_4_.^80,81^ It's crucial to recollect that the (002) peak of GO did not show up during chemical reduction with ascorbic acid, but a broad peak about 24.6° between 20° and 30° did show up, suggesting that the Fe_3_O_4_ nanoparticles were successfully coated on the graphene surface. The lack of a distinct peak at about 10° could potentially be attributed to the exfoliation of graphite oxide's layered structure. The partial rearranging of the delaminated graphene layers to produce a crystalline structure could be the reason for the large peak at around 24.6°.^78,82^

Using a superconducting quantum inference device, the magnetic characteristics of Fe_3_O_4_/GA were determined at ambient temperature with an applied magnetic field ranging from −10 000 to 10 000 Oe. The magnetization curve *versus* applied magnetic field in Fig. 1b illustrates the ferromagnetic characteristic of Fe_3_O_4_/GA. The hysteresis curve of the material is shown in Fig. 1b. Magnetic iron oxide nanoparticles have a saturation magnetism of *M*_s_ = 20.66 emu g^−1^. It proves that the magnetism of the Fe_3_O_4_/GA material is relatively high and can quickly separate from the sample solution through an external magnet, which can be used to recover materials in the field of environmental treatment promptly.

**Fig. 2:**
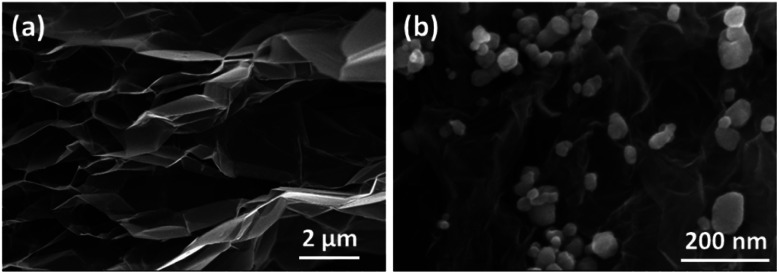
The SEM images of GO (a) and Fe_3_O_4_/GA (b).

The SEM images shown in Fig. 2 indicates that the GO material (Fig. 2a) has a moderately porous base without many voids. The morphology changed after incorporating magnetic iron oxide into graphene and changing the state from hydrogel to aerogel. The Fe_3_O_4_/GA material has many pores and magnetic iron oxide particles with particles size of less than 100 nm dispersed on the surface of transparent graphene layers. A thin-film interconnected porous 3D graphene is depicted in the presented SEM images of GO (Fig. 2a) and Fe_3_O_4_/GA (Fig. 2b), and Fe_3_O_4_ nanoparticles are uniformly distributed on the surface of the ultra-thin graphene layer.

**Fig. 3:**
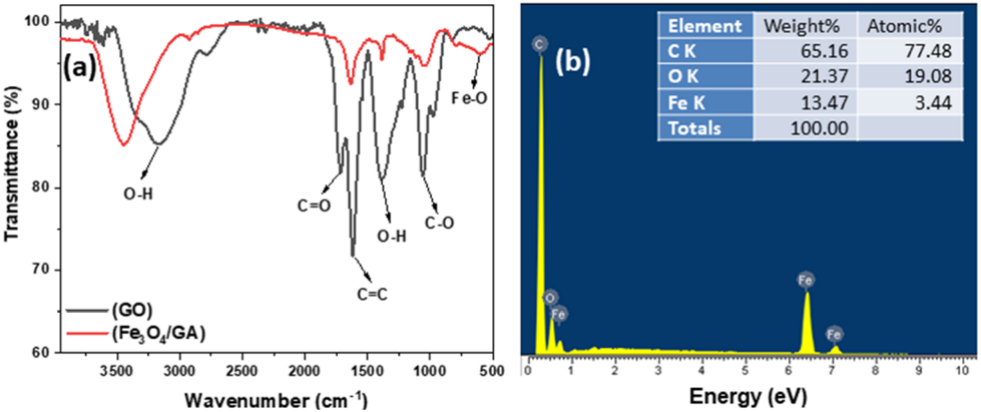
FTIR spectra (a) and EDX spectra (b) of Fe_3_O_4_/GA.

The samples characterized by FTIR infrared spectra shown in Fig. 3a are the corresponding GO, Fe_3_O_4_/GA spectra. In the region, 3500 cm^−1^ can be said to be the O–H group.^82^ The adsorption peaks at 1720 cm^−1^ correspond to the carbonyl functional groups at GO sheets' edges (COOH and C=O).^79^ The C=C vibrations of the graphene framework are thought to be responsible for the peak at 1620 cm^−1^. At 1410 cm^−1^ and 1060 cm^−1^, C–O vibrations in hydroxyl or epoxy groups were detected. One thing to notice is that, in comparison to the intensities in GO, all of the peak intensities at the vibrations of the oxygen functional groups in Fe_3_O_4_/GA are lower. This suggests that while the decrease of GO with ascorbic acid was successful, it was not total. In addition, because Fe_3_O_4_ covers the GO surface, the reduction process still needs to be completed. The Fe–O vibration of Fe_3_O_4_ has a distinctive vibration of approximately 590 cm^−1^, especially in the FTIR spectrum. The intensity of oxygen-containing groups decreased for Fe_3_O_4_/GA, suggesting partial reduction of GO to create rGO. At 590 cm^−1^, the Fe–O group appeared, signifying the bonding of ferromagnetic nanoparticles to the GA structure. The EDX spectra of Fe_3_O_4_/GA are shown in Fig. 3b. The presence of C, O, and Fe components in the mixture was confirmed by EDX spectra used to determine the elemental composition of Fe_3_O_4_/GA. Their existence suggests that the resultant goods are highly pure or do not have any significant sources of contaminants (<0.5% – EDX technical limit of measurement).

**Fig. 4:**
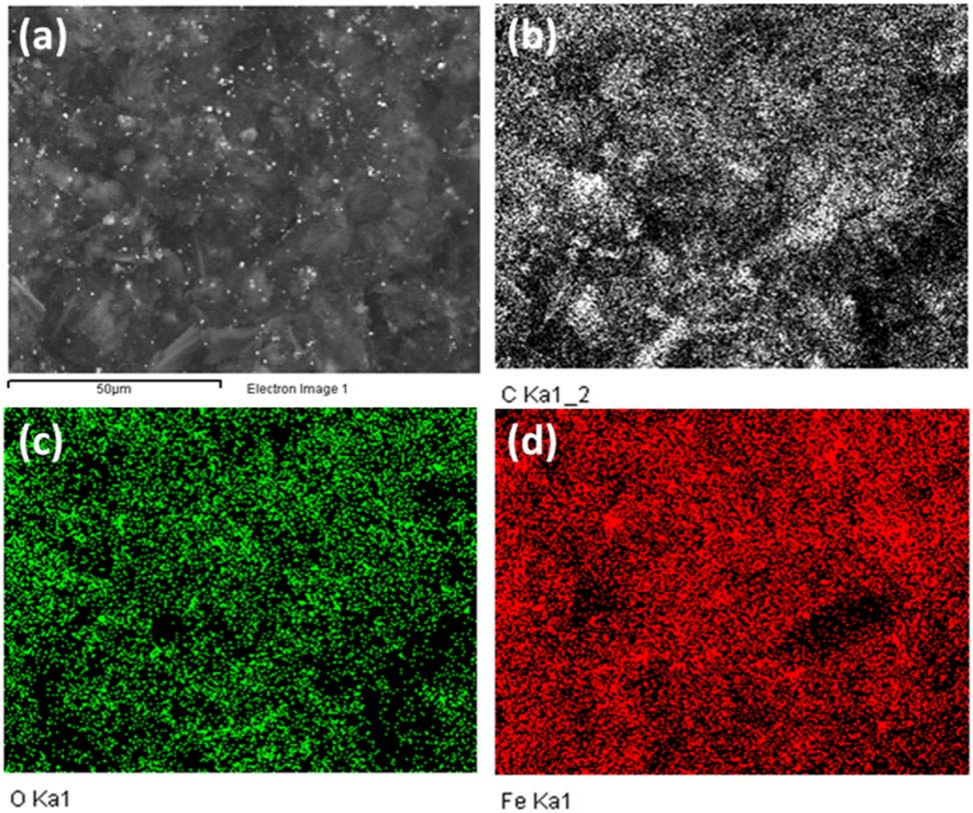
EDX mapping spectra of Fe_3_O_4_/GA with SEM image (a), C (b), O (c), and Fe (d) element distribution.

The EDX mapping analysis was employed to confirm the distribution of the Fe_3_O_4_ nanoparticles on the surface of GA. The results is shown in Fig. 4. It can be obvious that the Fe element are evenly distributed on the surface of the material. EDX spectrum analysis of Fe_3_O_4_/GA material in which the composition of elements C, O, and Fe are 65.16%, 21.37% and 13.47%, respectively. Fe_3_O_4_ nanoparticle distribution on the carbon aerogel's surface is demonstrated by the Fe_3_O_4_/GA's EDX mapping spectrum. The reduction process also took place as a result of the material's surface having a lower fraction of O atoms.

**Fig. 5:**
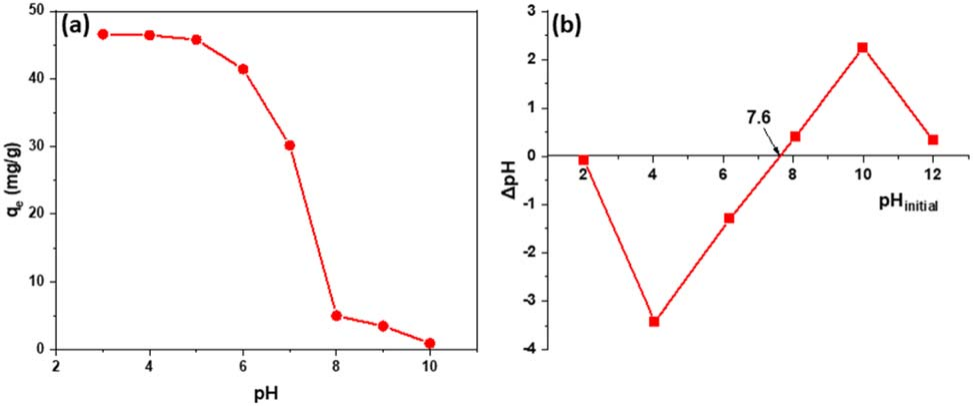
pH's impact on Fe_3_O_4_/GA's 2,4-D adsorption capacity (a) and isoelectric point (pH_pzc_) (b).

The results show that the material has good adsorption capacity in acidic environments around pH 3–5 and almost does not adsorb 2,4-D in alkaline environments around pH ≥ 8. The reason is when pH > pH_pzc_ (7.6) Fig. 5b, the alkaline environment on the material's surface will have a negative charge, adversely affecting the 2,4-D adsorption of Fe_3_O_4_/GA. In the pH range < pH_pzc_, the material surface will be positively charged. 2,4-D exists mainly in ionic form, so it is beneficial for adsorption. In addition, in an alkaline environment, there is competition for the adsorption of OH^−^, so the adsorption capacity of the material was reduced.

**Fig. 6:**
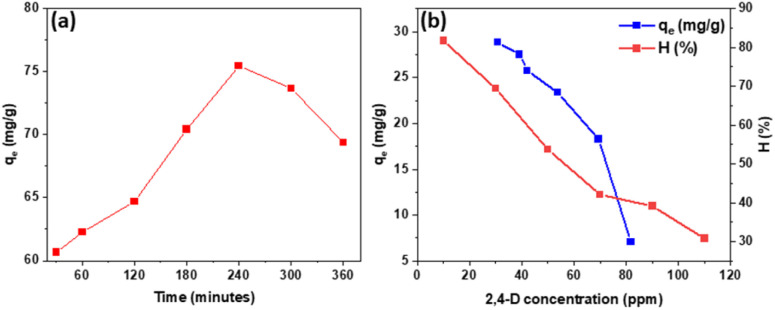
2,4-D adsorption load (a), efficiency and capacity (b) of Fe_3_O_4_/GA.

The findings demonstrated that at 240 minutes, the Fe_3_O_4_/GA's adsorption load peaked at 75.46 mg g^−1^ (Fig. 6a). Fig. 6b shows the effect of the 2,4-D concentrations on the adsorption efficiency. It can be seen that the adsorption efficiency is higher than 50% with 2,4-D concentrations of from 10 to 50 mg L^−1^. The adsorption efficiency decreased significantly when the, 4-D concentrations increases from 50 to 110 mg L^−1^. At first, the surface and capillaries are empty, and not much surface space is occupied. Furthermore, 2,4-D molecules are easily able to bind and penetrate. When the surface area occupied is large enough, the adsorption process takes place slowly and tends to increase slowly. Thus, the 2,4-D concentrations in range of 10 to 50 mg L^−1^ are considered appropriate concentration for the removal of 2,4-D by the Fe_3_O_4_/GA nanocomposite.

**Fig. 7:**
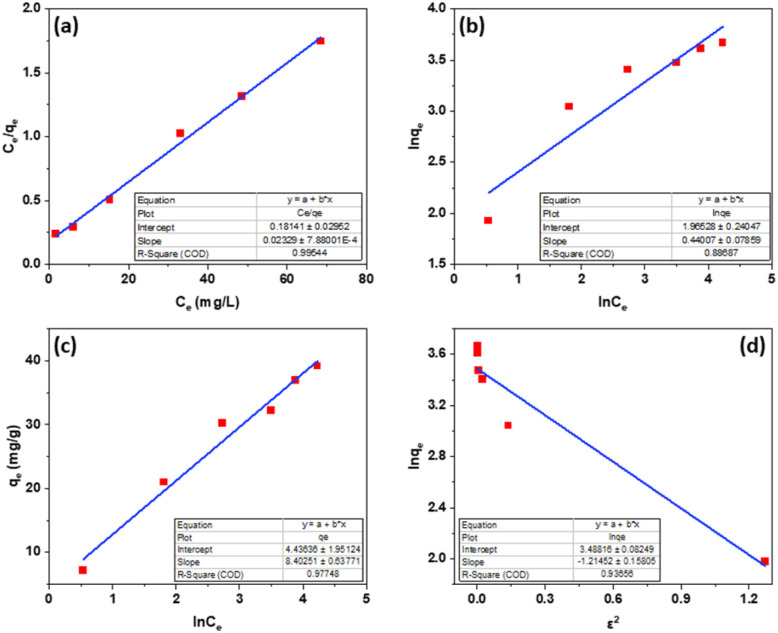
Fe_3_O_4_/GA linear 2,4-D adsorption curves by Langmuir (a), Freundlich (b), Temkin (c), and Dubinin–Radushkevich (d).

From the Langmuir, Freundlich, Tempkin, Dubinin–Radushkevich isothermal adsorption linear models of the material described in Fig. 7. We have calculated the parameters of the 2,4-D adsorption models of material Fe_3_O_4_/GA as shown in Table 2. According to the Freundlich model, *K*_F_ coefficient = 7.1371 and 1/*n* = 0.4401. The *K*_F_ value further demonstrates the high 2,4-D adsorption ability of Fe_3_O_4_/GA. The mode of the adsorption process is chemical contact, as evidenced by the low 1/*n* ratio. The 2,4-D adsorption process on Fe_3_O_4_/GA preferentially follows the Langmuir, Freundlich, and Temkin isotherm models, as seen from the regression coefficient *R*^2^ values found in the four models. The adsorption process is monolayer and chemical, as indicated by the significant regression coefficient of 0.9954. The experimental data show how well the Langmuir adsorption isotherm model describes the 2,4-D adsorption of Fe_3_O_4_/GA. Following the Langmuir model, the maximum adsorption capacity of Fe_3_O_4_/GA for 2,4-D was determined to be 42.918 mg g^−1^ (*R*^2^ = 0.9954). Adsorption occurs on the adsorbent material's surface with modest contact forces since its surface is homogeneous and contains many pores. The Freundlich equation's value of *n* > 1 denotes a favorable adsorption process. A weak contact between the adsorbents and the adsorbent is indicated by the Temkin constant, *b*_T_ = 0.2911, as shown in Table 2. Fe_3_O_4_/GA's physical adsorption of 2,4-D is supported in part by the force of contact between hydrogen bonds and π–π interactions. The 2,4-D adsorption process does not adhere to the Dubinin–Radushkevich model, as indicated by the correlation coefficient *R*^2^. We can get the adsorption energy value of 2,4-D on Fe_3_O_4_/GA (*E*) as 0.6416 kJ mol^−1^ < 8 kJ mol^−1^ using the Dubinin–Radushkevich model. That means adsorption takes place according to physical mechanisms.

**Table 2:** 2,4-D adsorption models' parameters of Fe_3_O_4_/GA

Isotherm models	Parameters	
Langmuir	*K*_L_ (L mg^−1^)	0.1284
	*q*_max_ (mg g^−1^)	42.918
	*R* ^2^	0.9954
Freundlich	*K*_F_ (mg g^−1^) (L mg^−1^)^1/*n*^	7.1371
	1/*n*	0.4401
	*R* ^2^	0.8869
Temkin	*K*_T_	6.6458
	*b*_T_ (kJ mol^−1^)	0.2911
	*R* ^2^	0.9775
Dubinin–Radushkevich	*q*_m_ (mg g^−1^)	37.921
	*β*	−1.2145
	*R* ^2^	0.9366
	*E* (kJ mol^−1^)	0.6416

**Fig. 8:**
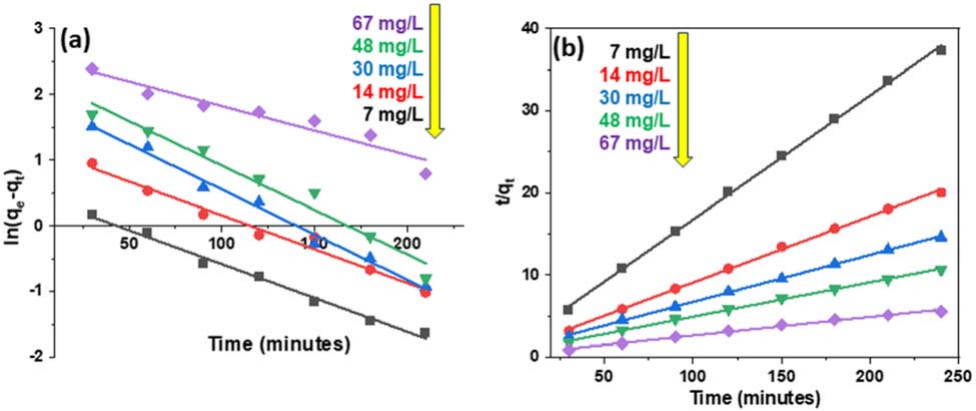
Pseudo-first-order (a) and second-order (b) kinetic models of Fe_3_O_4_/GA adsorption of 2,4-D.

Based on first- and second-order models, the kinetics of 2,4-D adsorption on Fe_3_O_4_/GA were constructed at 2,4-D concentrations of 7, 14, 30, 48, and 67 mg L^−1^, as illustrated in Fig. 8. Table 2 summarizes the adsorption rate constant and adsorption capacity determined using the first- and second-order apparent adsorption kinetic linear equations. The findings demonstrate that the second-order apparent kinetic model is consistent with the 2,4-D adsorption process on Fe_3_O_4_/GA.

The following formula represents the substance's adsorption rate in the pseudo-second-order kinetic model: *v* = *k*_2_*q*_e_^2^ (mg g^−1^ min^−1^) is shown in Table 3. When the adsorbent concentration is high, the amount of contact between the toxicant and the adsorbent surface increases. Therefore, the efficiency and adsorption rate increase.

**Table 3:** Some features of putative first- and second-order kinetic models that differ from the 2,4-D experiment

*C*_0_ (mg L^−1^)	*q*_e,exp_ (mg L^−1^)	Pseudo-first-order			Pseudo-second-order			
		*k*_1_ (L h^−1^)	*q*_e,cal_ (mg g^−1^)	*R* ^2^	*k*_2_ (g mg^−1^ h^−1^)	*q*_e,cal_ (mg g^−1^)	*R* ^2^	*v* (mg g^−1^ h^−1^)
7	6.435	0.0103	1.564	0.9912	0.0137	6.618	0.9990	0.600
14	11.982	0.0103	3.283	0.9785	0.0064	12.361	0.9986	0.978
30	16.411	0.0137	6.869	0.9874	0.0033	17.422	0.9992	1.002
48	22.521	0.0130	9.681	0.9654	0.0024	23.810	0.9980	1.984
67	43.110	0.0075	13.070	0.9260	0.0013	44.248	0.9929	2.545

According to the Langmuir model, the maximum adsorption capacity of 2.4-D by Fe_3_O_4_/GA was calculated to be 45.872 mg g^−1^, which is comparable to other absorbents that have been previously reported (Table 4). Fe_3_O_4_/GA, on the other hand, has a more affordable production method and is readily scalable. According to this study, Fe_3_O_4_/GA may be applied more frequently in real-world scenarios than other adsorbents.

**Table 4:** The 2,4-D adsorption capability of different adsorbents

Adsorbent	2,4-D adsorption capacity, mg g^−1^	References
Graphene oxide/Fe_3_O_4_	5.62	83
Graphene/Fe_3_O_4_	32.31	84
Polyaniline–Fe_3_O_4_	60.97	85
Polypyrrole–Fe_3_O_4_	96.15	86
UiO-66-NH_2_	72.99	87
Black carbon	64.00	88
Organo-palygorskite OP2CEC	42.00	89
Fe_3_O_4_/GA	42.918	*This study*

## Conclusions

In summary, the hydrothermal approach has been effectively applied in the synthesis of Fe_3_O_4_/GA. The magnetic iron oxide nanoparticles are uniformly shaped, spherical, and have an average particle size of between 50 and 100 nm. They are uniformly distributed on the GO aerogel basis. The material's magnetism reaches 20.66 emu g^−1^. Fe_3_O_4_/GA has an excellent adsorption capacity and efficiency, according to research examining factors impacting its ability to remove 2,4-D from water. The ideal pH range for the 2,4-D additive is 5–6, and it takes 240 minutes to equilibrate. It climbs quickly at concentration ranges ≤50 mg L^−1^ and progressively falls when 2,4-D concentration >50 mg L^−1^. The acquired experimental values are in agreement with the 42.918 mg g^−1^ maximum adsorption capacity of the Langmuir, Freundlich, and Temkin isothermal adsorption theoretical model. A multimodal adsorption method, involving physical and chemical electrostatic interaction forces resulting from π–π interactions and hydrogen bonds, is employed to remove 2,4-dichlorophenoxyacetic acid from the material. The pseudo-second-order kinetic model describes the process kinetics. Using a magnet to extract the Fe_3_O_4_/GA from the solution is a practical and convenient way to utilize its strong magnetism.

## Data availability

Data for this article, including XRD, hysteresis curve, SEM, FTIR, EDX, adsorption isotherm and kinetic models are available at Open Science Framework at https://osf.io/hp9cu/.

## Author contributions

Thu Hang Thi Nguyen and Kim Thuy Nguyen set up the experiment, collected and analyzed the data. Bao Hung Le wrote the manuscript. Xuan Truong Nghiem provided the methodology and conceptualization. Duy Khiem Nguyen drafted the data and used the software to process and analyze the data. Duc Duong La reviewed and edited the manuscript. And Hoai Phuong Thi Nguyen supervised the authors in conducting the research and completing the manuscript.

## Conflicts of interest

There are no conflicts to declare.
